# Genetic contributions to autism spectrum disorder

**DOI:** 10.1017/S0033291721000192

**Published:** 2021-10

**Authors:** A. Havdahl, M. Niarchou, A. Starnawska, M. Uddin, C. van der Merwe, V. Warrier

**Affiliations:** 1 Nic Waals Institute, Lovisenberg Diaconal Hospital, Oslo, Norway; 2 Department of Mental Disorders, Norwegian Institute of Public Health, Oslo, Norway; 3 Department of Psychology, PROMENTA Research Center, University of Oslo, Oslo, Norway; 4 Vanderbilt Genetics Institute, Vanderbilt University Medical Center, TN, USA; 5 The Lundbeck Foundation Initiative for Integrative Psychiatric Research, iPSYCH, Denmark; 6 Department of Biomedicine, Aarhus University, Denmark; 7 Center for Genomics for Personalized Medicine, CGPM, and Center for Integrative Sequencing, iSEQ, Aarhus, Denmark; 8 College of Medicine, Mohammed Bin Rashid University of Medicine and Health Sciences, Dubai, UAE; 9 Stanley Center for Psychiatric Research, The Broad Institute of MIT and Harvard, MA, USA; 10 Department of Psychiatry, Autism Research Centre, University of Cambridge, UK

**Keywords:** Autism, common variation, epigenetics, genetics, heterogeneity, rare variation, transcriptomics

## Abstract

Autism spectrum disorder (autism) is a heterogeneous group of neurodevelopmental conditions characterized by early childhood-onset impairments in communication and social interaction alongside restricted and repetitive behaviors and interests. This review summarizes recent developments in human genetics research in autism, complemented by epigenetic and transcriptomic findings. The clinical heterogeneity of autism is mirrored by a complex genetic architecture involving several types of common and rare variants, ranging from point mutations to large copy number variants, and either inherited or spontaneous (*de novo*). More than 100 risk genes have been implicated by rare, often *de novo*, potentially damaging mutations in highly constrained genes. These account for substantial individual risk but a small proportion of the population risk. In contrast, most of the genetic risk is attributable to common inherited variants acting *en masse*, each individually with small effects. Studies have identified a handful of robustly associated common variants. Different risk genes converge on the same mechanisms, such as gene regulation and synaptic connectivity. These mechanisms are also implicated by genes that are epigenetically and transcriptionally dysregulated in autism. Major challenges to understanding the biological mechanisms include substantial phenotypic heterogeneity, large locus heterogeneity, variable penetrance, and widespread pleiotropy. Considerable increases in sample sizes are needed to better understand the hundreds or thousands of common and rare genetic variants involved. Future research should integrate common and rare variant research, multi-omics data including genomics, epigenomics, and transcriptomics, and refined phenotype assessment with multidimensional and longitudinal measures.

## Definition of autism

Kanner defined autism in 1943 with detailed case descriptions of children showing social aloofness, communication impairments, and stereotyped behaviors and interests, often accompanied by intellectual disability (ID) (Kanner, 1943). A year later, Asperger independently published an article on children presenting marked difficulties in social communication and unusually circumscribed and intense interests, despite advanced intellectual and language skills (Asperger, 1944). Three decades later, Wing and Gould united Asperger and Kanner's descriptions and conceptualized a spectrum of autistic conditions (Wing and Gould, 1978, 1979).

The onset of autism is during the first years of life, although symptoms may not be fully apparent or recognized until later (American Psychiatric Association, 2013). Autism is a heterogeneous and complex group of conditions with considerable variation in core symptoms, language level, intellectual functioning, and co-occurring psychiatric and medical difficulties. Subtype diagnoses such as childhood autism and Asperger's syndrome were previously used to specify more homogeneous presentations, but were unstable over time within individuals and used unreliably by clinicians (Lord et al., 2020). Current editions of the major diagnostic manuals have replaced the subtypes with an overarching autism spectrum disorder diagnosis and instead require specification of key sources of heterogeneity; language level, intellectual functioning, and co-occurring conditions (APA, 2013; World Health Organization, 2018).

## Epidemiology

Prevalence estimates of autism have steadily increased from less than 0.4% in the 1970s to current estimates of 1–2% (Fombonne, 2018; Lyall et al., 2017). The increase is largely explained by broadening diagnostic criteria to individuals without ID and with milder impairments, and increased awareness and recognition of autistic traits (Lord et al., 2020; Taylor et al., 2020). There are marked sex and gender differences in autism (Halladay et al., 2015; Warrier et al., 2020). The male-to-female ratio is approximately 4:1 in clinical and health registry cohorts but closer to 3:1 in general population studies with active case-finding (Loomes, Hull, & Mandy, 2017) and 1–2:1 in individuals with moderate-to-severe ID (Fombonne, 1999; Yeargin-Allsopp et al., 2003). The mechanisms underlying the sex difference are mostly unknown, and hypotheses include a female protective effect (aspects of the female sex conferring resilience to risk factors for autism), prenatal steroid hormone exposure, and social factors such as underdiagnosis and misdiagnosis in women (Ferri, Abel, & Brodkin, 2018; Halladay et al., 2015).

Co-occurring conditions are the rule rather than the exception, estimated to affect at least 70% of people with autism from childhood (Lai et al., 2019; Simonoff et al., 2008). Common co-occurring conditions include attention-deficit hyperactivity disorder (ADHD), anxiety, depression, epilepsy, sleep problems, gastrointestinal and immune conditions (Davignon, Qian, Massolo, & Croen, 2018; Warrier et al., 2020). There is an elevated risk of premature mortality from various causes, including medical comorbidities, accidental injury, and suicide (Hirvikoski et al., 2016).

Autism is also associated with positive traits such as attention to detail and pattern recognition (Baron-Cohen & Lombardo, 2017; Bury, Hedley, Uljarević, & Gal, 2020). Further, there is wide variability in course and adulthood outcomes with regard to independence, social relationships, employment, quality of life, and happiness (Howlin & Magiati, 2017; Mason et al., 2020; Pickles, McCauley, Pepa, Huerta, & Lord, 2020). Rigorous longitudinal studies and causally informative designs are needed to determine the factors affecting developmental trajectories and outcomes.

### Environmental factors

Twin studies suggest that 9–36% of the variance in autism predisposition might be explained by environmental factors (Tick, Bolton, Happé, Rutter, & Rijsdijk, 2016). There is observational evidence for association with pre- and perinatal factors such as parental age, asphyxia-related birth complications, preterm birth, maternal obesity, gestational diabetes, short inter-pregnancy interval, and valproate use (Lyall et al., 2017; Modabbernia, Velthorst, & Reichenberg, 2017). Mixed results are reported for pregnancy-related nutritional factors and exposure to heavy metals, air pollution, and pesticides, while there is strong evidence that autism risk is unrelated to vaccination, maternal smoking, or thimerosal exposure (Modabbernia et al., 2017). It is challenging to infer causality from observed associations, given that confounding by lifestyle, socioeconomic, or genetic factors contributes to non-causal associations between exposures and autism. Many putative exposures are associated with parental genotype (e.g. obesity, age at birth) (Gratten et al., 2016; Taylor et al., 2019a, Yengo et al., 2018), and some are associated both with maternal and fetal genotypes (e.g. preterm birth) (Zhang et al., 2017). Studies triangulating genetically informative designs are needed to disentangle these relationships (Davies et al., 2019; Leppert et al., 2019; Thapar & Rutter, 2019).

## Twin and pedigree studies

In 1944, Kanner noted that parents shared common traits with their autistic children, introducing the ‘broader autism phenotype’ (i.e. sub-threshold autistic traits) and recognizing the importance of genetics (Harris, 2018; Kanner, 1944). Thirty years later, twin studies revolutionized the field of autism research (Ronald & Hoekstra, 2011).

**Fig. 1.**
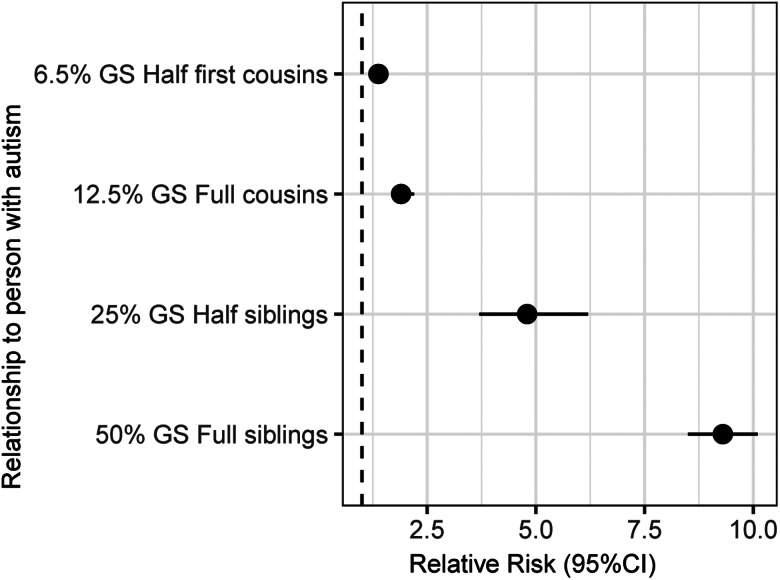
Relative risk of autism by degree of relatedness with a person with autism. Relative risk for full and half siblings, and full cousins was provided in Hansen et al. (2019). Relative risk for half first cousins was estimated based on Xie et al. (2019). GS, genome shared.

Twin studies were the first to demonstrate the heritability of autism. In 1977, the first twin-heritability estimate was published, based on a study of 10 dizygotic (DZ) and 11 monozygotic (MZ) pairs (Folstein & Rutter, 1977). Four out of the 11 MZ pairs (36%) but none of the DZ pairs were concordant for autism. Subsequently, over 30 twin studies have been published, further supporting the high heritability of autism (Ronald & Hoekstra, 2011). A meta-analysis of seven primary twin studies reported that the heritability estimates ranged from 64% to 93% (Tick et al., 2016). The correlations for MZ twins were at 0.98 [95% confidence interval (CI) 0.96–0.99], while the correlations for DZ twins were at 0.53 (95% CI 0.44–0.60) when the autism prevalence rate was assumed to be 5% (based on the broader autism phenotype) and increased to 0.67 (95% CI 0.61–0.72) when the prevalence was 1% (based on the stricter definition) (Tick et al., 2016). Additionally, family studies have found that the relative risk of a child having autism relates to the amount of shared genome with affected relatives (Fig. 1) (Bai et al., 2019; Constantino et al., 2013; Georgiades et al., 2013; Grønborg, Schendel, & Parner, 2013; Risch et al., 2014; Sandin et al., 2014).

Early twin and pedigree studies demonstrated that the biological relatives of individuals with autism who did not meet the criteria for an autism diagnosis themselves commonly showed elevated autistic traits such as communication and social interaction difficulties (Le Couteur et al., 1996), indicating that the heritability is not restricted to the traditional diagnostic boundaries of autism. Twin studies also indicate that although social communication and repetitive behavior trait dimensions each show strong heritability, there is a limited genetic correlation between them (e.g. for a review, see Ronald & Hoekstra, 2011). Further, twin studies have found substantial genetic overlap between autistic traits and symptoms of other psychiatric conditions, including language delay (e.g. Dworzynski et al., 2008), ID (e.g. Nishiyama et al., 2009), ADHD (e.g. Ronald, Edelson, Asherson, & Saudino, 2010), and anxiety (e.g. Lundström et al., 2011) (for a review, see Ronald & Hoekstra, 2014). Moreover, twin and family studies indicate that the sibling recurrence rate of autism is lower in female than male siblings (Palmer et al., 2017; Werling & Geschwind, 2015), suggesting the female protective effect hypothesis as a potential explanation for the male preponderance in the diagnosis of autism. The hypothesis was supported by results showing that the siblings of autistic females had a higher likelihood of high autistic trait scores and autism than the siblings of autistic males (Ferri et al., 2018; Palmer et al., 2017; Robinson, Lichtenstein, Anckarsäter, Happé, & Ronald, 2013), consistent with females having a higher liability threshold.

## Genetics

Genetic variants differ in the frequency at which they occur in the population (e.g. rare *v.* common), the type (i.e. SNPs/CNVs/translocations and inversions/indels), and whether they are inherited or *de novo*. Here, we summarize the findings on genetic risk for autism from linkage and candidate gene studies, common and rare genetic variation studies, epigenomics, and transcriptomics. A glossary of important terms is in Box 1.

Box 1. Glossary

**Candidate gene association study:** A study that examines the association between a phenotype and a genetic variant chosen *a priori* based on knowledge of the gene's biology or functional impact.

**Complex trait:** A trait that does not follow Mendelian inheritance patterns, but is likely the result of multiple factors including a complex mixture of variation within multiple genes.

**Copy number variant (CNV):** Deletion or duplication of large genomic regions.

***de novo* mutation:** A mutation that is present in the offspring but is either absent in parents or is present only in parental germ cells.

**DNA methylation (DNAm):** Epigenetic modification of DNA characterized by the addition of a methyl group (-CH_3_) to the 5^th^ position of the pyrimidine ring of cytosine base resulting in 5-methylcytosine (5mC).

**Epigenetics:** The science of heritable changes in gene regulation and expression that do not involve changes to the underlying DNA sequence.

**Epigenome-Wide Association Study (EWAS):** A study that investigates associations between DNA methylation levels quantified at tens/hundreds of thousands of sites across the human genome, and the trait of interest.

**Genome-Wide Association Study (GWAS):** A study scanning genome-wide genetic variants for associations with a given trait.

**Genetic correlation:** An estimate of the proportion of variance shared between two traits due to shared genetics.

**Heritability:** An estimate of the proportion of variation in a given trait that is due to differences in genetic variation between individuals in a given population.

**Heritability on the liability scale**: A heritability estimate adjusted for the population prevalence of a given binary trait, typically disorders.

**Genetic linkage studies:** A statistical method of mapping genes of heritable traits to their chromosomal locations by using chromosomal co-segregation with the phenotype.

**Mendelian inheritance:** When the inheritance of traits is passed down from parents to children and is controlled by a single gene for which one allele is dominant and the other recessive.

**Methylation Quantitative Trait Locus (mQTL):** A SNP at which genotype is correlated with the variation of DNA methylation levels at a nearby (*cis-*mQTL) or distal (*trans-*mQTL) site.

**Phenotype:** The observable characteristics of an individual.

**Polygenic risk score (PRS):** An estimate of an individual's genetic liability for a condition calculated based on the cumulative effect of many common genetic variants.

**Single nucleotide polymorphism (SNP):** A single base pair change that is common (>1%) in the population.

**Single nucleotide variant (SNV):** A variation in a single nucleotide without any limitation of frequency.

**SNP heritability:** The proportion of variance in a given phenotype in a population that is attributable to the additive effects of all SNPs tested. Typically, SNPs included have a minor allele frequency >1%.

### Linkage and candidate gene studies

Initial linkage studies were conducted to identify chromosomal regions commonly inherited in affected individuals. Susceptibility loci implicated a range of regions, but only two have been replicated (Ramaswami & Geschwind, 2018): at chromosome 20p13 (Weiss, Arking, Daly, & Chakravarti, 2009) and chromosome 7q35 (Alarcón, Cantor, Liu, Gilliam, & Geschwind, 2002). Lack of replication and inconsistent findings were largely due to low statistical power (Kim & Leventhal, 2015). Candidate gene association studies identified over 100 positional and/or functional candidate genes for associations with autism (Bacchelli & Maestrini, 2006). However, there was no consistent replication for any of these findings (Warrier, Chee, Smith, Chakrabarti, & Baron-Cohen, 2015), likely due to limitations in study design (e.g. low statistical power, population diversity, incomplete coverage of variation within the candidate genes, and false positives arising from publication bias) (Ioannidis, 2005; Ioannidis, Ntzani, Trikalinos, & Contopoulos-Ioannidis, 2001). The advancement of genome-wide association studies (GWAS) and next-generation sequencing techniques has significantly enhanced gene and variant discovery.

### Common genetic variation

**Fig. 2.**
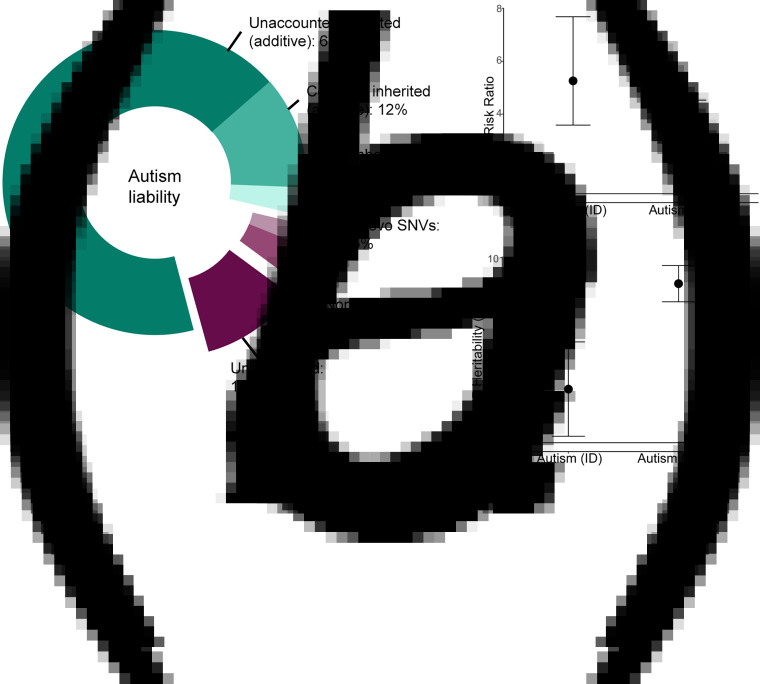
Variance explained by different classes of genetic variants in autism. (*a*) Donut chart of the variance explained by different classes of variants. The narrow-sense heritability (82.7%, Nordic average, shades of green) has been estimated using familial recurrence data from Bai et al. (2019). The total common inherited heritability (12%) has been estimated using LDSC-based SNP-heritability (additive) from Grove et al. (2019) and the total rare inherited heritability (3%) has been obtained from Gaugler et al. (2014). The currently unexplained additive heritability is thus 67.7% (total narrow-sense heritability minus common and rare inherited heritabilities combined). This leaves a total of 17.3% of the variance to shared and unique environmental estimates (Bai et al., 2019). The term environmental refers to non-additive and non-inherited factors that contribute to variation in autism liability. Of this, *de novo* missense and protein-truncating variants (Satterstrom et al., 2020) and variation in non-genic regions (An et al., 2018) together explain 2.5% of the variance. Whilst *de novo* variation can be inherited in some cases (germline mutation in the parent) and thus shared between siblings, it is unlikely that this will be shared by other related individuals, and thus unlikely to be included in the narrow-sense heritability in Bai et al. (2019). This is likely to be a lower-bound of the estimate as we have not included the variance explained by *de novo* structural variants and tandem repeats. Additionally, non-additive variation accounts for ~4% of the total variance (Autism Sequencing Consortium et al., 2019). Thus, ~11% of the total variance is currently unaccounted for, though this is likely to be an upper bound. (*b*) The variance explained is likely to change in phenotypic subgroups. For instance, the risk ratio for *de novo* protein-truncating variants in highly constrained genes (pLI > 0.9) is higher in autistic individuals with ID compared to those without ID (point estimates and 95% confidence intervals provided; Kosmicki et al., 2017). (*c*) Similarly, the proportion of the additive variance explained by common genetic variants is higher in autistic individuals without ID compared to autistic individuals with ID (Grove et al., 2019). Point estimates and 95% confidence intervals provided.

The SNP-heritability (proportion of variance attributed to the additive effects of common genetic variants) of autism ranges from 65% in multiplex families (Klei et al., 2012) to 12% in the latest Psychiatric Genomics Consortium GWAS (Fig. 2*a*) (Autism Spectrum Disorders Working Group of The Psychiatric Genomics Consortium, 2017; Grove et al., 2019). Variation is largely attributable to sample heterogeneity and differences in methods used to estimate SNP-heritability.

**Fig. 3.**
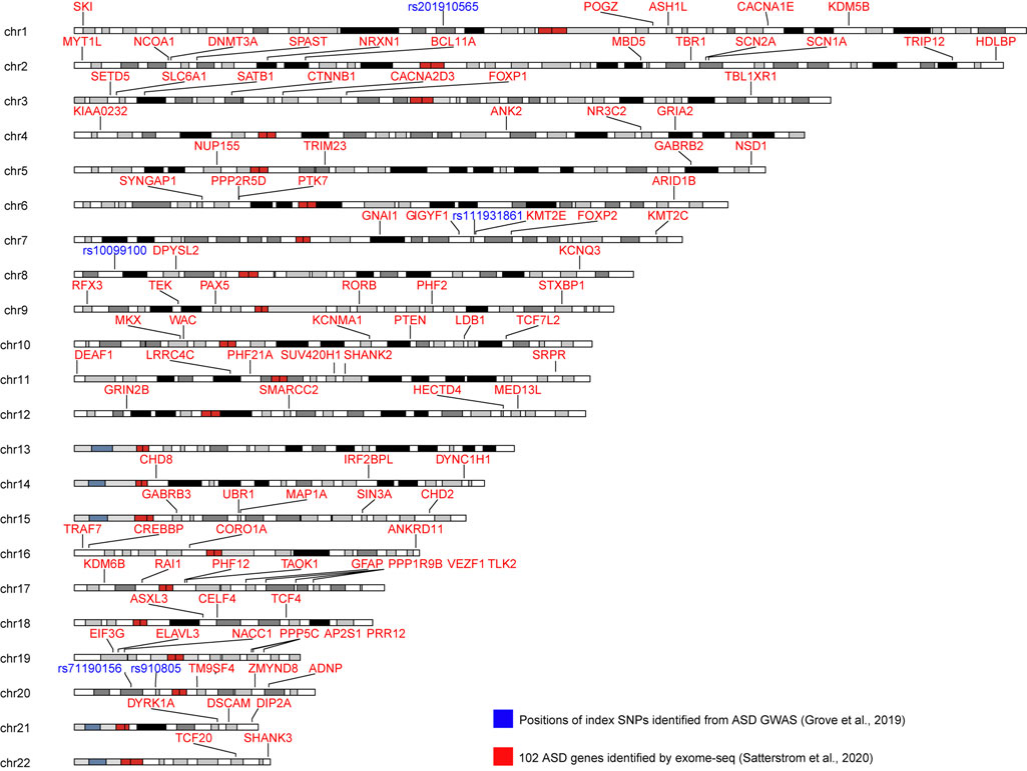
Karyogram showing the 102 genes implicated by rare variant findings at a false discovery rate of 0.1 or less (Satterstrom et al., 2020) and the five index SNPs identified in GWAS (Grove et al., 2019) of autism.

Early GWASs of autism were underpowered, partly due to overestimating potential effect sizes. Grove et al. (2019) conducted a large GWAS of autism combining data from over 18 000 autistic individuals and 27 000 non-autistic controls and an additional replication sample. They identified five independent GWAS loci (Fig. 3). Another recent study (Matoba et al., 2020) identified a further novel locus by meta-analyzing the results from Grove et al. (2019) with over 6000 case-pseudocontrol pairs from the SPARK cohort by employing a massively parallel reporter assay to identify a potential causal variant (rs7001340) at this locus which regulates *DDH2* in the fetal brain. The sample sizes are still relatively small compared to other psychiatric conditions (Schizophrenia Working Group of the Psychiatric Genomics Consortium, 2020; Howard et al., 2019), though ongoing work aims to double the sample size and identify additional loci.

Using genetic correlations and polygenic score analyses, studies have identified modest shared genetics between autism and different definitions of autistic traits in the general population (Askeland et al., 2020; Bralten et al., 2018; Robinson et al., 2016; Taylor et al., 2019*b*). There is some evidence for developmental effects, with greater shared genetics in childhood compared to adolescence (St Pourcain et al., 2018). These methods have also identified modest polygenic associations between autism and other neurodevelopmental and mental conditions such as schizophrenia, ADHD, and major depressive disorder, related traits such as age of walking, language delays, neuroticism, tiredness, and self-harm, as well as risk of exposure to childhood maltreatment and other stressful life events (Brainstorm Consortium et al., 2018; Bulik-Sullivan et al., 2015; Grove et al., 2019; Hannigan et al., 2020; Lee et al., [Bibr ref76][Bibr ref76]; Leppert et al., 2019; Cross-Disorder Group of the Psychiatric Genomics Consortium, 2013; Warrier & Baron-Cohen, 2019). Notably, autism is positively genetically correlated with measures of intelligence and educational attainment (EA) (Bulik-Sullivan et al., 2015; Grove et al., 2019), an observation supported by polygenic score association (Clarke et al., 2016). Polygenic Transmission Disequilibrium Tests have identified an over-transmission of polygenic scores for EA, schizophrenia, and self-harm from parents to autistic children, but an absence of such over-transmission to non-autistic siblings (Warrier & Baron-Cohen, 2019; Weiner et al., 2017), suggesting that these genetic correlations are not explained by ascertainment biases or population stratification. However, a genetic correlation does not necessarily imply a causal relationship between the two phenotypes and may simply index biological pleiotropy. Causal inference methods such as Mendelian randomization can be used to disentangle such relationships (Davies et al., 2019; Pingault et al., 2018).

The relatively low SNP-heritability in autism compared to other psychiatric conditions may partly be due to phenotypic heterogeneity. In an attempt to reduce phenotypic heterogeneity, Chaste et al. (2015) identified 10 phenotypic combinations to subgroup autistic individuals. Family-based association analyses did not identify significant loci, and SNP-heritability for the subgroups was negligent. It is unclear if reducing phenotypic heterogeneity increases genetic homogeneity, and investigating this in larger samples is warranted. Another study identified no robust evidence of genetic correlation between social and non-social (restricted and repetitive behavior patterns) autistic traits (Warrier et al., 2019). A few studies have investigated the common variant genetic architecture of social and non-social autistic traits in individuals with autism (Alarcón et al., 2002; Cannon et al., 2010; Cantor et al., 2018; Lowe, Werling, Constantino, Cantor, & Geschwind, 2015; Tao et al., 2016; Yousaf et al., 2020) and in the general population (St Pourcain et al., 2014; Warrier et al., 2018, 2019), but replication of the identified loci is needed.

Diagnostic classification is another source of heterogeneity: SNP-heritability of Asperger's syndrome (ICD-10 diagnosis) was twice (0.097 ± 0.001) that of childhood autism and unspecified pervasive developmental disorders (Grove et al., 2019) [due to overlap in subtype diagnoses, a hierarchy was used: childhood autism>atypical autism>Asperger's syndrome>unspecified subtypes (Grove et al., 2019)]. Supporting this, polygenic scores for intelligence and EA had larger loadings in the Asperger's syndrome and childhood autism subgroups compared to other subgroups (Grove et al., 2019). Additionally, the SNP-heritability of autism (all subtypes) without co-occurring ID diagnosis (0.09 ± 0.005) was three times that of autism with ID (Grove et al., 2019) (Fig. 2*c*).

### Rare genetic variation

Rare genetic variants confer significant risk in the complex etiology of autism. They are typically non-Mendelian, with substantial effect sizes and low population attributable risk. It is estimated that ~10% of autistic individuals have been diagnosed with an identifiable rare genetic syndrome characterized by dysmorphia, metabolic, and/or neurologic features (Carter & Scherer, 2013; Tammimies et al., 2015). Associated syndromes include the 15q11-q13 duplication of the Prader-Willi/Angelman syndrome, fragile X syndrome, 16p11.2 deletion syndrome, and 22q11 deletion syndrome (Sztainberg & Zoghbi, 2016). Prevalence estimates for autism vary widely between genetic syndromes; for example, 11% in 22q11.2 deletion syndrome and 54% in Cohen's syndrome (Richards, Jones, Groves, Moss, & Oliver, 2015). Of note, estimating the prevalence of autism in the context of genetic syndromes is complex (Havdahl et al., 2016; Richards et al., 2015).

The rate of gene discovery in autism is a linear function of increasing sample size (De Rubeis et al., 2014). Early studies implicated nine genes in the first 1000 autism cases (Neale et al., 2012; Sanders et al., 2012), increasing to 27 and 33 associated genes from separate analyses of Simons Simplex Collection and Autism Sequencing Consortium (ASC) samples (De Rubeis et al., 2014; Iossifov et al., 2014). Integrating these samples using the TADA framework implicated a total of 65 autism genes (Sanders et al., 2015).

The MSSNG initiative analyzed whole genomes from 5205 individuals (*N*_cases_ = 2636), and identified 61 autism-risk genes, of which 18 were new candidates (Yuen et al., 2017). More recently, the largest whole-exome sequencing analysis to date conducted by the ASC (*N* = 35 584, *N*_cases_ = 11 986) identified 102 autism-associated genes (Fig. 3), many of which are expressed during brain development with roles in the regulation of gene expression and neuronal communication (Satterstrom et al., 2020). Rare CNVs and SNVs associated with autism have pleiotropic effects, thus increasing the risk for other complex disorders such as schizophrenia, ADHD, ID, and epilepsy (Gudmundsson et al., 2019; Satterstrom et al., 2019, 2020).

#### CNVs

CNVs can impact one or multiple genes and can occur at common or rare frequencies in a population. All CNVs associated with autism have been rare. Recurrent CNVs are among the most convincing rare inherited risk variations for autism, and have a prevalence of about 3% in affected patients (Bourgeron, 2016). In comparison, approximately 4–10% of autistic individuals have *de novo* deletions or duplications (Bourgeron, 2016; Pinto et al., 2010; Sebat et al., 2007) frequently mapped to established risk loci 1q21.1, 3q29, 7q11.23, 15q11.2-13, and 22q11.2 (Sanders et al., 2015). A higher global frequency of *de novo* CNVs is observed in idiopathic autism cases from simplex families (10%) compared to multiplex families (2%) and controls (1%) (Halladay et al., 2015; Itsara et al., 2010; Sebat et al., 2007). Inherited CNVs can be present in unaffected siblings and parents, suggesting a model of incomplete penetrance dependent on the dosage sensitivity and function of the gene(s) they affect (Vicari et al., 2019).

#### SNVs

Damaging SNVs include nonsense, frameshift, and splice site mutations (collectively referred to as protein-truncating variants, or PTVs), and missense variants. Rare inherited variants have a smaller average effect size and reduced penetrance compared to *de novo* pathogenic mutations. Early studies on whole exomes from trios established a key role for *de novo* germline mutations in autism. Whilst analysis in smaller sample sizes indicated only modest increase in *de novo* mutation rates in autism cases (Neale et al., 2012), the rate rose significantly in excess of expectation as the sample size increased (De Rubeis et al., 2014; Iossifov et al., 2014). Most recently, the ASC observed a 3.5-fold case enrichment of damaging *de novo* PTVs and a 2.1-fold enrichment for damaging *de novo* missense variants (Satterstrom et al., 2020), concluding that all exome *de novo* SNVs explain 1.92% of the variance in autism liability (Satterstrom et al., 2020) (Fig. 2*a*).

Comparatively, the ASC discovered a 1.2-fold enrichment of rare inherited damaging PTVs in cases compared to unaffected siblings (Satterstrom et al., 2020). Similarly, recent whole-genome analysis found no excess of rare inherited SNVs, and no difference in the overall rate of these variants in affected subjects compared to unaffected siblings (Ruzzo et al., 2019).

#### New advancements

It is estimated that *de novo* mutations in protein-coding genes contribute to risk in ~30% of simplex autism cases (Yuen et al., 2017; Zhou et al., 2019). However, recent work has also shown that *de novo* mutations in non-coding regions of the genome (particularly gene promoters) contribute to autism (An et al., 2018; Zhou et al., 2019). Adapting machine learning techniques may be key to providing novel neurobiological insights to the genetic influences on autism in the future (An et al., 2018; Ruzzo et al., 2019; Zhou et al., 2019). Additionally, rare tandem repeat expansions in genic regions are more prevalent among autism cases than their unaffected siblings, with a combined contribution of ~2.6% to the risk of autism (Trost et al., 2020).

#### Common and rare variant interplay

The largest component of genetic risk is derived from common variants of additive effect with a smaller contribution from *de novo* and rare inherited variation (Fig. 2*a*) (de la Torre-Ubieta, Won, Stein, & Geschwind, 2016; Gaugler et al., 2014). Notably, *KMT2E* was implicated in both the latest GWAS (Grove et al., 2019) and exome sequencing (Satterstrom et al., 2020) analyses. It is hypothesized that common genetic variation in or near the genes associated with autism influences autism risk, although current sample sizes lack the power to detect the convergence of the two (Satterstrom et al., 2020).

Whilst higher SNP-heritability is observed in autistic individuals without ID (Fig. 2*b*), *de novo* PTVs in constrained genes are enriched in autistic individuals with ID (Fig. 2*a*). However, the genetic architecture of autism is complex and diverse. For example, common genetic variants also contribute to risk in autistic individuals with ID and in autistic individuals carrying known large-effect *de novo* variants in constrained genes (Weiner et al., 2017). Furthermore, an excess of disruptive *de novo* variants is also observed in autistic individuals without co-occurring ID compared to non-autistic individuals (Satterstrom et al., 2020).

### Epigenetics

DNA methylation (DNAm), an epigenetic modification, allows for both genetic and environmental factors to modulate a phenotype (Martin & Fry, 2018; Smith et al., 2014). DNAm affects gene expression, regulatory elements, chromatin structure, and alters neuronal development, functioning, as well as survival (Kundaje et al., 2015; Lou et al., 2014; Peters et al., 2015; Sharma, Klein, Barboza, Lohdi, & Toth, 2016; Yu et al., 2012; Zlatanova, Stancheva, & Caiafa, 2004). Additionally, putative prenatal environmental risk factors impact the offspring's methylomic landscape (Anderson, Gillespie, Thiele, Ralph, & Ohm, 2018; Cardenas et al., 2018; Joubert et al., 2016), thus providing a plausible molecular mechanism to modulate the neurodevelopmental origins of autism.

Autism Epigenome-Wide Association Study (EWAS) meta-analysis performed in blood from children and adolescents from SEED and SSC cohorts (*N*_cases_ = 796, *N*_controls_ = 858) identified seven differentially methylated positions (DMPs) associated (*p* < 10 × 10^−05^) with autism, five of them also reported to have brain-based autism associations. The associated DMPs annotated to *CENPM*, *FENDRR*, *SNRNP200*, *PGLYRP4*, *EZH1*, *DIO3*, and *CCDC181* genes, with the last site having the largest effect size and the same direction of association with autism across the prefrontal cortex, temporal cortex, and cerebellum (Andrews et al., 2018). The study reported moderate enrichment of methylation Quantitative Trait Loci (mQTLs) among the associated findings, suggesting top autism DMPs to be under genetic control (Andrews et al., 2018). These findings were further extended by the MINERvA cohort that added 1263 neonatal blood samples to the meta-analysis. The SEED-SSC-MINERvA meta-EWAS identified 45 DMPs, with the top finding showing the consistent direction of association across all three studies annotated to *ITLN1* (Hannon et al., 2018). The MINERvA sample was also used for EWAS of autism polygenic score, hypothesizing that the polygenic score-associated DNAm variation is less affected by environmental risk factors, which can confound case–control EWAS. Elevated autism polygenic score was associated with two DMPs (*p* < 10 × 10^−06^), annotated to *FAM167A*/*C8orf12* and *RP1L1*. Further Bayesian co-localization of mQTL results with autism GWAS findings provided evidence that several SNPs on chromosome 20 are associated both with autism risk and DNAm changes in sites annotated to *KIZ*, *XRN2*, and *NKX2-4* (Hannon et al., 2018). The mQTL effect of autism risk SNPs was corroborated by an independent study not only in blood, but also in fetal and adult brain tissues, providing additional evidence that autism risk variants can act through DNAm to mediate the risk of the condition (Hammerschlag, Byrne, Bartels, Wray, & Middeldorp, 2020).

Since autism risk variants impact an individual's methylomic landscape, studies that investigate DNAm in the carriers of autism risk variants are of interest to provide insight into their epigenetic profiles. A small blood EWAS performed in 52 cases of autism of heterogeneous etiology, nine carriers of 16p11.2del, seven carriers of pathogenic variants in *CHD8*, and matched controls found that DNAm patterns did not clearly distinguish autism of the heterogeneous etiology from controls. However, the homogeneous genetically-defined 16p11.2del and *CHD8^+/−^* subgroups were characterized by unique DNAm signatures enriched in biological pathways related to the regulation of central nervous system development, inhibition of postsynaptic membrane potential, and immune system (Siu et al., 2019). This finding highlights the need to combine genomic and epigenomic information for a better understanding of the molecular pathophysiology of autism.

It must be noted that a very careful interpretation of findings from peripheral tissues is warranted. DNAm is tissue-specific and therefore EWAS findings obtained from peripheral tissues may not reflect biological processes in the brain. Using the mQTL analytical approach may reduce this challenge, as mQTLs are consistently detected across tissues, developmental stages, and populations (Smith et al., 2014). However, not all mQTLs will be detected across tissues and will not necessarily have the same direction of effect (Smith et al., 2014). Therefore, it is recommended that all epigenetic findings from peripheral tissues are subjected to replication analyses in human brain samples, additional experimental approaches, and/or Mendelian randomization to strengthen causal inference and explore molecular mediation by DNAm (Walton, Relton, & Caramaschi, 2019).

EWASs performed in post-mortem brains have typically been conducted using very small sample sizes, due to limited access to brain tissue (Ladd-Acosta et al., 2014; Nardone et al., 2014). One of the largest autism EWAS performed in post-mortem brains (43 cases and 38 controls) identified multiple DMPs (*p* < 5 × 10^−05^) associated with autism (31 DMPs in the prefrontal cortex, 52 in the temporal cortex, and two in the cerebellum) (Wong et al., 2019), and autism-related co-methylation modules to be significantly enriched for synaptic, neuronal, and immune dysfunction genes (Wong et al., 2019). Another post-mortem brain EWAS reported DNAm levels at autism-associated sites to resemble the DNAm states of early fetal brain development (Corley et al., 2019). This finding suggests an epigenetic delay in the neurodevelopmental trajectory may be a part of the molecular pathophysiology of autism.

Overall, methylomic studies of autism provide increasing evidence that common genetic risk variants of autism may alter DNAm across tissues, and that the epigenetic dysregulation of neuronal processes can contribute to the development of autism. Stratification of study participants based on their genetic risk variants may provide deeper insight into the role of aberrant epigenetic regulation in subgroups within autism.

### Transcriptomics

#### Transcriptomics of peripheral tissues

Gene expression plays a key role in determining the functional consequences of genes and identifying genetic networks underlying a disorder. One of the earliest studies on genome-wide transcriptome (Nishimura et al., 2007) investigated blood-derived lymphoblastoid cells gene expression from a small set of males with autism (*N* = 15) and controls. Hierarchical clustering on microarray expression data followed by differentially expressed gene (DEG) analysis revealed a set of dysregulated genes in autism compared to controls. This approach was adopted (Luo et al., 2012) to investigate DEGs in a cohort of 244 families with autism probands (index autism case in a family) known to carry *de novo* pathogenic or variants of unknown significance and discordant sibling carriers of non-pathogenic CNVs. From genome-wide microarray transcriptome data, this study identified significant enrichment of outlier genes that are differentially expressed and reside within the proband rare/*de novo* CNVs. Pathway enrichment of these outlier genes identified neural-related pathways, including neuropeptide signaling, synaptogenesis, and cell adhesion. Distinct expression changes of these outlier genes were identified in recurrent pathogenic CNVs, i.e. 16p11.2 microdeletions, 16p11.2 microduplications, and 7q11.23 duplications. Recently, multiple independent genome-wide blood-derived transcriptome analysis (Filosi et al., 2020; Lombardo et al., 2018; Tylee et al., 2017) showed the efficiency of detecting dysregulated genes in autism, including aberrant expression patterns of long non-coding RNAs (Sayad, Omrani, Fallah, Taheri, & Ghafouri-Fard, 2019).

#### Transcriptomics of post-mortem brain tissue

Although blood-derived transcriptome can be feasible to study due to easy access to the biological specimen, blood transcriptome results are not necessarily representative of the transcriptional machinery in the brain (GTEx Consortium, 2017). Hence, it is extremely hard to establish a causal relationship between blood transcriptional dysregulations and phenotypes in autism. A landmark initiative by Allen Brain Institute to profile human developing brain expression patterns (RNA-seq) from post-mortem tissue enabled neurodevelopmental research to investigate gene expression in the brain (Sunkin et al., 2013). Analyzing post-mortem brain tissue, multiple studies identified dysregulation of genes at the level of gene exons impacted by rare/*de novo* mutations in autism (Uddin et al., 2014; Xiong et al., 2015), including high-resolution detection of exon splicing or novel transcript using brain tissue RNA sequencing (RNA-seq). High-resolution RNA-seq enabled autism brain transcriptome analysis on non-coding elements, and independent studies identified an association with long non-coding RNA and enhancer RNA dysregulation (Wang et al., 2015; Yao et al., 2015; Ziats & Rennert, 2013).

**Fig. 4.**
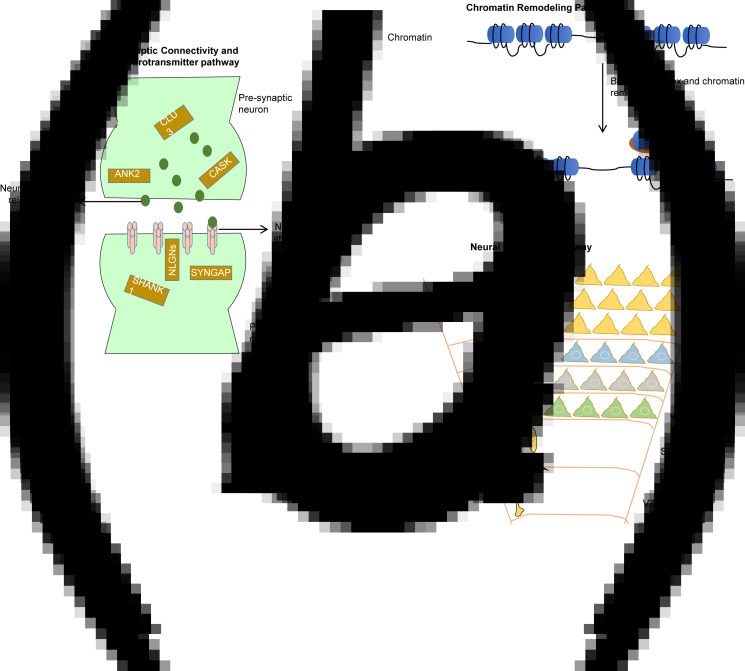
Most commonly reported three pathways (Ayhan & Konopka, 2019; Gordon et al., 2019; Voineagu et al., 2011) associated with autism. (*a*) The synaptic connectivity and neurotransmitter pathway involves genes (yellow rectangular box) within presynaptic and postsynaptic neurons. Neurotransmitter transport through numerous receptors is an essential function of this pathway; (*b*) the chromatin remodeling pathway involves binding of remodeling complexes that initiate the repositioning (move, eject, or restructure) of nucleosomes that potentially can disrupt gene regulation; and (*c*) the neural projection pathway [adapted from Greig, Woodworth, Galazo, Padmanabhan, & Macklis (2013)] involves the projection of neural dendrite into distant regions and the migration of neuronal cells through ventricular (VZ) and subventricular zones (SVZ) into the different cortical layers (I-VI).

Although it is difficult to access post-mortem brain tissue from autistic individuals, studies of whole-genome transcriptome from autism and control brains have revealed significantly disrupted pathways (Fig. 4) related to synaptic connectivity, neurotransmitter, neuron projection and vesicles, and chromatin remodeling pathways (Ayhan & Konopka, 2019; Gordon et al., 2019; Voineagu et al., 2011). Recently, an integrated genomic study also identified from autism brain tissue a component of upregulated immune processes associated with hypomethylation (Ramaswami et al., 2020). These reported pathways are in strong accordance with numerous independent autism studies that integrated genetic data with brain transcriptomes (Courchesne, Gazestani, & Lewis, 2020; Uddin et al., 2014; Yuen et al., 2017). A large-scale analysis of brain transcriptome from individuals with autism identified allele-specific expressions of genes that are often found to be impacted by pathogenic *de novo* mutations (Lee et al., 2019*a*). The majority of the studies are in consensus that genes that are highly active during prenatal brain development are enriched for clinically relevant mutations in autism (Turner et al., 2017; Uddin et al., 2014; Yuen et al., 2017). Recently, a large number (4635) of expression quantitative trait loci were identified that were enriched in prenatal brain-specific regulatory regions comprised of genes with distinct transcriptome modules that are associated with autism (Walker et al., 2019).

#### Single-cell transcriptomics

Recent advancement of single-cell transcriptomics enables the detection of cell types that are relevant to disorder etiology. A recent case–control study conducted single-cell transcriptomics analysis on 15 autism and 16 control cortical post-mortem brain tissues generating over 100 000 single-cell transcriptomics data (Velmeshev et al., 2019). Cell-type analysis revealed dysregulations of a specific group of genes in cortico-cortical projection neurons that correlate with autism severity (Velmeshev et al., 2019). Deciphering cell-type identification has future implications, in particular for the implementation of precision medicine. However, single-cell technology is at very early stages of development and computationally it is still very complex to classify cell-type identity.

The emergence of CRISPR/Cas9 genome editing technology can potentially become an effective tool in future therapeutics of genetic conditions associated with autism. Although introducing and reversing DNA mutation is becoming a mature technology within *in vitro* systems, much work needs to be done for *in vivo* use of genome editing. Single-cell OMICs is another emerging field that has the potential to decipher developmental (spatio-temporally) brain cell types that are associated with autism. Identifying cell clusters and defining cell identity is a major computational challenge. Artificial intelligence can significantly improve these computational challenges to identify the molecular associations of autism at the single-cell level.

### Clinical and therapeutic implications

In some, but not all, best practice clinical guidelines, genetic tests such as fragile X testing, chromosomal microarray, and karyotype testing are part of the standard medical assessment in a diagnostic evaluation of autism to identify potentially etiologically relevant rare genetic variants (Barton et al., 2018). The guidelines vary with respect to whether genetic testing is recommended for all people with autism, or based on particular risk factors, such as ID, seizures, or dysmorphic features. The DSM-5 diagnosis of autism includes a specifier for associated genetic conditions (APA, 2013). Although genetic test results may not usually have consequences for treatment changes, the results could inform recurrence risk and provide families with access to information about symptoms and prognosis. In the future, gene therapy, CRISPR/Cas9, and genome editing technologies may lead to the gene-specific design of precision medicine for rare syndromic forms of autism (Benger, Kinali, & Mazarakis, 2018; Gori et al., 2015).

Given that a substantial proportion of the genetic liability to autism is estimated to be explained by the cumulative effect of a large number of common SNPs, polygenic scores have gained traction as potential biomarkers. However, the predictive ability of polygenic scores from the largest autism GWAS to date is too low to be clinically useful. The odds ratio when comparing the top and bottom polygenic score decile groups is only 2.80 (95% CI 2.53–3.10) (Grove et al., 2019). Additionally, polygenic scores based on the samples of European ancestry do not translate well in populations with diverse ancestry (Palk, Dalvie, de Vries, Martin, & Stein, 2019).

Genetic testing can in the future become useful for informing screening or triaging for diagnostic assessments or identifying who may be more likely to respond to which type of intervention (Wray et al., 2021). Genetics may also help identify individuals with autism who are at a high risk of developing co-occurring physical and mental health conditions or likely to benefit from treatments of such conditions. A top research priority for autistic people and their families is addressing co-occurring mental health problems (Autistica, 2016), which may sometimes be the primary treatment need as opposed to autism *per se*. Genomics may also be helpful to repurpose existing treatments and better identify promising treatments. There are active clinical trials to repurpose drugs in autism (Hong & Erickson, 2019). Moreover, genetics can be used to identify social and environmental mediating and moderating factors (Pingault et al., 2018), which could inform interventions to improve the lives of autistic people.

Notably, there are important ethical challenges related to clinical translation of advances in genetics, including concerns about discriminatory use, eugenics concerning prenatal genetic testing, and challenges in interpretation and feedback (Palk et al., 2019). People with autism and their families are key stakeholders in genetic studies of autism and essential to include in discussions of how genetic testing should be used.

## Conclusions and future directions

Recent large-scale and internationally collaborative investigations have led to a better understanding of the genetic contributions to autism. This includes identifying the first robustly associated common genetic variants with small individual effects (Grove et al., 2019) and over 100 genes implicated by rare, mostly *de novo*, variants of large effects (Sanders et al., 2015; Satterstrom et al., 2020). These and other findings show that the genetic architecture of autism is complex, diverse, and context-dependent, highlighting a need to study the interplay between different types of genetic variants, identify genetic and non-genetic factors influencing their penetrance, and better map the genetic variants to phenotypic heterogeneity within autism.

Immense collaborative efforts are needed to identify converging and distinct biological mechanisms for autism and subgroups within autism, which can in turn inform treatment (Thapar & Rutter, 2020). It is crucial to invest in multidimensional and longitudinal measurements of both core defining traits and associated traits such as language, intellectual, emotional, and behavioral functioning, and to collaboratively establish large omics databases including genomics, epigenomics, transcriptomics, proteomics, and brain connectomics (Searles Quick, Wang, & State, 2020). Indeed, large-scale multi-omic investigations are becoming possible in the context of large population-based family cohorts with rich prospective and longitudinal information on environmental exposures and developmental trajectories of different neurodevelopmental traits. Finally, novel methods (Neumeyer, Hemani, & Zeggini, 2020) can help investigate causal molecular pathways between genetic variants and autism and autistic traits.
